# Acute Urinary Retention and Guillain-Barré Syndrome: A Zebra or a Horse

**DOI:** 10.1016/j.acepjo.2025.100313

**Published:** 2026-01-08

**Authors:** Martin Klinkhammer, Navjot Kaur, James Wyant

**Affiliations:** 1 Department of Emergency Medicine, Eastern Virginia Medical School, Norfolk, Virginia, USA; 2 Department of Neurology, Eastern Virginia Medical School, Norfolk, Virginia, USA

**Keywords:** Guillain-Barré Syndrome, cauda equina, observation medicine, acute urinary retention

## Abstract

Acute immune-mediated polyneuropathies are grouped under the umbrella term Guillain-Barré syndrome (GBS). GBS can often present with variable clinical findings. Typical features of GBS include a progressive, symmetric muscle weakness and absent deep tendon reflexes. This case report describes a confusing presentation in a patient who had been having preceding back pain prior to the onset of a worsening back pain associated with autonomic symptoms of GBS with urinary retention. This caused initial diagnostic confusion with cauda equina syndrome being the leading diagnostic possibility until ruled out with magnetic resonance imaging. The case demonstrates the importance of not anchoring completely on the initial diagnostic probability but on continuing the workup of acute urinary retention associated with other neurologic symptoms as cauda equina was effectively ruled out. The case also highlights the sometimes problematic errors that can occur with observation medicine.

## 1 Introduction

Guillain-Barré syndrome (GBS) is an acute immune-mediated polyradiculoneuropathy. It most commonly presents with progressive, symmetric muscle weakness with absent deep tendon reflexes on examination. The typical clinical course presents with progressive symptoms over a 2-week course, although the nadir of symptoms can go out as late as 4 weeks from presentation. Many aspects of the disease are not completely understood.

### 1.1 Case

A 25-year-old man with no significant past medical history presented to the emergency department (ED) for a second visit due to lower back pain. He stated that he first began to have back pain several months earlier and had presented to this ED 6 weeks earlier. During that visit, he had a reported unremarkable neurologic examination, and lumbar and thoracic spine X-rays were obtained, which were normal. He stated that since that visit, he has followed up with his primary care doctor several times including earlier that same week, and no further imaging or lab testing was done. However, for the last 2 weeks, he has noted worsening back pain and difficulty in controlling his urine as well as his stool. At times, he has awakened at night having lost bladder control and wetting the bed. He also has had intermittent difficulty controlling his bowels. Lastly, he noted a tingling burning pain occasionally shooting into his legs during these same 2 weeks. He additionally noted occasional tingling in his hands but felt that that would resolve if he shook them out.

Upon examination, he was well appearing. His neurologic examination demonstrated an equivocal straight leg raise test bilaterally. He had good strength in all extremities to resistance testing with a grossly normal gait. Sensation was intact in all extremities. His rectal examination demonstrated diminished tone, and he had absent reflexes bilaterally at the knees and ankles.

A postvoid bladder ultrasound was done, shown in the Figure, demonstrating that he was retaining 386 mL of urine.

**Figure:**
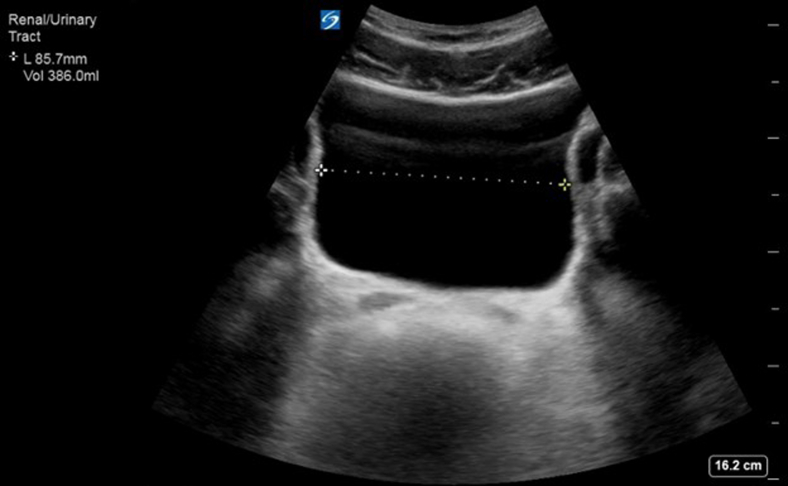
Ultrasound of postvoid bladder.

Given the concern for possible cauda equina syndrome or other lower spinal cord acute pathology, in consultation with neurosurgery by phone, an magnetic resonance imaging (MRI) of his thoracic and lumbar spines was ordered. Because the results of the MRI may necessitate transfer to an alternate neurosurgical center, he was placed in ED observation to obtain the MRIs. This resulted in him having to be turned over to an incoming provider. The plan at the time of turnover was that, should his MRIs demonstrate a neurosurgical issue, he would need to be transferred. If the MRIs were negative, the plan was for hospital admission for neurology consultation.

The MRIs of his thoracic and lumbar spine were read as follows: minimal degenerative changes, no evidence of herniation or significant stenosis, and no abnormality that would correlate with a cauda equina syndrome or thoracic myelopathy.

Unfortunately, there was an error in communication, and the second provider reexamined the patient and discharged him home to follow up with neurology as an outpatient, which in our community usually takes months.

The following day, the original provider was now working at a different ED across town. When he got to work, he reviewed the chart to see if the MRIs had indeed confirmed cauda equina. He was taken aback that the patient was discharged home. He called the patient, who confirmed that he was still quite concerned about his urinary incontinence, which he had experienced again that night. He was referred back to the ED, this time at the second hospital, and had a lumbar puncture performed and a neurology consultation. On neurology consultation, it was noted that he had subtle weakness in his lower extremities not been detected by the previous ED physicians. Given his absence of reflexes, the diagnosis of GBS was made, and he was started on treatment with intravenous immunoglobulin (IVIG) for 5 days. With this treatment, all symptoms other than some mild remaining back pain resolved and he was discharged home on hospital day 6 with no further issues. The patient has remained relatively symptom-free at the time of this report.

## 2 Discussion

Acute urinary retention in a previously healthy 25-year-old man, particularly when accompanied by other neurologic deficits, should be taken seriously and worked up extensively. GBS typically presents with progression of symptoms over 0 to 4 weeks and has a nadir of symptoms by 4 weeks into the course. This patient’s original back pain had been present for several months, but his urinary and bowel incontinence had only been present for 2 weeks at the time of presentation. Thus, the back pain complaint was at least partly a distractor and not actually the primary symptom contributing to the patient’s ultimate diagnosis. The patient was likely having an alternate cause of back pain such as a strained muscle and then additionally developed GBS. As far as GBS triggers, in this case, the patient had been vaccinated against influenza approximately 2 months previously, but he also had had multiple upper respiratory as well as diarrheal illnesses in the months preceding due to frequent exposure to young children in his home.

Although perhaps poorly understood by some providers, back pain can be a common part of the presentation of GBS^1^^,^^2^ and is typically felt in the interscapular area or in a radicular pattern, such as was the case in this presentation. Back pain can be one of the presenting symptoms of GBS in up to 62% of cases.^2^^,^^3^ Additionally, although muscle weakness is more commonly a chief presenting feature, urinary retention can also be a presenting symptom of GBS.^4^

In cases that continue to progress past 4 weeks, the diagnosis is more likely to be chronic inflammatory demyelinating polyradiculoneuropathy (CIDP). The difference is important as the treatment for CIDP is steroids, whereas the treatment for GBS is either IVIG or plasma exchange therapy (PLEX), and steroids have been found to be ineffective.^5^ In this case, the patient’s urinary symptoms and weakness had only progressed for the 2 previous weeks. On lumbar puncture, he did not have an albuminocytologic dissociation as is often seen in GBS, but this can be the case in up to 30% of cases.^6^ His response with complete recovery to IVIG strongly suggests that his diagnosis was GBS as a somewhat unusual presentation.

Although observation medicine certainly has a place in reducing health care costs, cases such as this highlight how it can often be fraught with errors. Fortunately, in this case, when the initial diagnostic plan was not followed, the patient was reachable the following day, such that he was ultimately able to be treated in a timely manner. The efficaciousness of IVIG in the treatment of GBS is known to be time sensitive, with greatest benefit in the first 2 weeks and little utility after 4 weeks.^1^ There is the potential that had the patient not been reachable, he likely would not have received treatment in time to make an impact, potentially leading to longstanding urinary incontinence, as well as the potential for significant progression of his symptoms.

## Funding and Support

By *JACEP Open* policy, all authors are required to disclose any and all commercial, financial, and other relationships in any way related to the subject of this article as per ICMJE conflict of interest guidelines (see www.icmje.org). The authors have stated that no such relationships exist.

## Conflict of Interest

All authors have affirmed they have no conflicts of interest to declare.
